# School nurses’ perspectives on key features of a mobile app to support early identification of adolescent mental health problems: a qualitative study

**DOI:** 10.1186/s12912-026-05302-7

**Published:** 2026-08-29

**Authors:** Elin Holmgren Celander, N. Gårevik, Zarina Nahar Kabir, Lisa Ring, Lise-Lott Rydström

**Affiliations:** 1 Department of Neurobiology, Care Sciences and Society (NVS), Karolinska Institutet, Fack, Huddinge, 23400, SE-141 83 Sweden; 2 Department of Health Sciences, Swedish Red Cross University, P.O. Box 1059, Huddinge, SE-141 21 Sweden

**Keywords:** Adolescent mental health, School nursing, Digital health, Mobile applications, mHealth, Qualitative research, Help-seeking, Thematic analysis

## Abstract

**Background:**

Adolescent mental ill health is increasing globally and in Sweden, with rising levels of stress, anxiety, depression, and sleep problems. Many adolescents do not seek help due to stigma, fear of judgement, and limited mental health literacy. School nurses play a key role in early identification and preventive support. Digital tools may complement relational nursing practices and lower thresholds for help-seeking, but little is known about what features school nurses consider feasible and useful in their daily practice.

**Objective:**

To explore school nurses’ perspectives on key features and content of a mobile app intended to support early identification and management of mental health problems among high school students.

**Methods:**

An exploratory qualitative study was conducted using semi-structured interviews with ten school nurses working in upper secondary schools. Interviews were held via Zoom, transcribed verbatim, and analysed using reflexive thematic analysis (RTA). COREQ guidelines informed the study’s design and reporting.

**Results:**

Two overarching themes emerged. First, Fostering mental wellbeing: the school nurse as a trusted ally in a complex world highlighted how early identification often relies on relational continuity, trust, and interpretation of indirect indicators such as somatic complaints or behavioural changes. Second, Nurturing minds: the digital dance of support and solitude reflected nurses’ belief that a mobile app could support time management, self-regulation, psychoeducation, and accessible communication. Feasibility, however, depended on safeguarding confidentiality, maintaining professional boundaries, preventing workload escalation, and managing the “anonymity paradox,” in which anonymity can both facilitate disclosure and hinder safe escalation.

**Conclusions:**

School nurses perceive that a mobile app could complement face-to-face care by supporting early identification and self-management among adolescents. Successful implementation requires aligning digital features with relational nursing practice, ensuring ethical governance, and integrating clear escalation pathways. Thoughtfully designed mHealth solutions have potential to strengthen preventive mental health support within school health services.

**Clinical trial number:**

Not applicable.

**Supplementary Information:**

The online version contains supplementary material available at https://doi.org/10.1186/s12912-026-05302-7.

## Abstract (significance)

What does this paper contribute to the wider global clinical community?

1. Even though school nurses already use different tools and methods to address mental ill health, they still require more information, tools and methods to be able to meet the needs of today's adolescents

2. A digital intervention in the form of a mobile app with various features, including for example interaction with students, self-management and information about habits was considered a helpful way forward.

3. A digital intervention addressing mental ill health is not a novelty but, as far as we know, there is no other digital intervention available for school nurses in their work with mental ill health that includes interactions with the student and other features to support mental health.

**Supplementary Information:**

The online version contains supplementary material available at https://doi.org/10.1186/s12912-026-05302-7.

## Implications for the professionand/or patient care

Relational and observational competence as core nursing practice. The findings underscore that school nurses’ capacity to identify mental health concerns often rests on relational continuity and skilled interpretation of indirect indicators, such as somatic complaints and behavioural changes. This implies that school health services should safeguard time and organisational conditions that enable trust-building, informal encounters, and reflective clinical judgement. For students, such relationally grounded practice facilitates earlier recognition of distress and increases the likelihood of timely, appropriate support. Holistic and collaborative early intervention. Mental health concerns among students emerge from a convergence of academic pressures, lifestyle factors, family dynamics, and broader societal stressors. Consequently, school nursing practice must be supported by structured collaboration with teachers and parents and by assessment approaches that integrate psychological, social, and physical dimensions of wellbeing. From a person-centred patient-care perspective, coordinated and context-sensitive interventions improve continuity, address underlying contributors to distress, and reinforce preventive efforts. Hybrid models of care require clear boundaries and ethical governance. The potential integration of mobile apps highlights the need for carefully designed hybrid care models in school health services. For the profession, this entails establishing clear boundaries for availability, safeguarding confidentiality, and ensuring that digital tools complement rather than replace face-to-face care. For students, digitally supported self-management and communication can lower thresholds to help-seeking and improve accessibility, provided that personal relationships, safety mechanisms, and escalation pathways remain central to care delivery.

## Reporting method

This study adheres to the COREQ guidelines for the reporting of qualitative research [1]. Patient or Public Contribution. No patient or public contribution.

## Introduction

Adolescence is a formative developmental period during which emotional regulation, problem-solving abilities, and everyday health behavioural take shape in interaction with school, family, peers, and digital environments, all of which influence present and future wellbeing [2, 3]. Globally, approximately one in seven adolescents experience mental ill health, with anxiety and depression being the most prevalent conditions [2, 3]. In Sweden, self-reported mental health problems among young people have increased steadily since the 1980s, with rising levels of stress, worry, sleep difficulties, and psychosomatic symptoms [4, 5]. Despite this growing burden, many adolescents do not seek professional help. Well-documented barriers include stigma, fear of judgement, limited mental health literacy, and a preference for self-reliance [6–8].

Within the Swedish student healthcare system, school nurses play a key role in health promotion and early identification of mental health concerns, mandated by the Education Act [9]. Their ongoing presence in school settings enables relational continuity, trust-building, low-threshold access, and timely observation of changes that may signal distress. Mental health problems in adolescents often present indirectly through somatic complaints, behavioural changes, or decreased school engagement [5]. School nurses’ clinical judgement and relational knowledge are therefore essential for recognising emerging concerns. At the same time, Swedish regulatory frameworks, including the Patient Safety Act [10], require that confidentiality and professional responsibility be upheld in all interactions.

Digital mental health interventions—such as mobile apps, web-based programs, and hybrid models—have expanded rapidly. Systematic reviews report positive effects on anxiety, depression, distress, mental health knowledge, stigma reduction, and help-seeking when interventions include components such as psychoeducation, self-monitoring, and interactive support [11–13]. School-based digital interventions have also shown promise in increasing reach and engagement, improving awareness, reducing symptoms, and supporting adolescents who may otherwise avoid traditional services [14–16]. However, concerns regarding confidentiality, data protection, sustained usability, ethical governance, and long-term outcomes remain central to implementation efforts [17, 18].

For school nursing specifically, digital tools are most feasible when understood as *relational technologies*—supports that enhance rather than replace face-to-face relational work. School nurses describe clear potential benefits, including lower thresholds for help-seeking, support for self-regulation (sleep, activity, stress), structured follow-up, and more accessible communication. Yet they also highlight significant challenges, including workload implications, unclear expectations of availability, confidentiality risks, and the “anonymity paradox,” in which anonymity may encourage disclosure but hinder safe escalation in cases of risk [15–17, 19].

Despite growing interest in digital mental health solutions, little is known about how tools should be designed with and for school nurses within the Swedish student health context to ensure acceptability, ethical integrity, and practical feasibility.

In this study, the proposed mobile app refers to a digital support tool primarily intended for use by upper secondary school students. The app was envisioned as a complement to existing school health services and could include features such as psychoeducation, self-monitoring of wellbeing, self-management support, and communication with school nurses when appropriate. The intention is not to replace face-to-face contact but to facilitate early identification, help-seeking, and access to support.

### Aim

To explore school nurses’ perspectives on key features and content of a mobile app, as a formative pre-study to guide the design and development of a digital intervention for early identification and management of mental health problems among high school students.

## Method

### Design

An exploratory qualitative study was conducted using semi-structured interviews.

#### Study setting and recruitment

School principals were contacted via purposive and snowball sampling to reach upper secondary schools in Stockholm and nearby counties that varied in organisational and demographic characteristics. After principals granted permission, school nurses received written study information and informed consent forms. Ten school nurses agreed to participate.

#### Sampling strategy and sample size rationale

A purposive sampling strategy was used to recruit school nurses with relevant experience in identifying and supporting students with mental health concerns. To capture differences across organisational and demographic contexts, a maximum-variation sampling logic was applied, ensuring diversity in school type (public/independent), socioeconomic area, and programme orientation.

Consistent with reflexive thematic analysis (RTA) [20], sample adequacy in RTA is judged by thematic sufficiency—the point at which additional interviews are unlikely to offer new interpretative insights. Based on methodological guidance for RTA, a sample of approximately 8–14 participants were anticipated to provide sufficient analytic depth. Recruitment ceased at ten interviews when ongoing analysis indicated that further data collection would not generate new, meaningful contributions to the developing themes.

#### Inclusion criteria

Participants were required to be employed as school nurses in upper secondary schools and to hold specialist qualifications in paediatric nursing, school health nursing, or primary health care nursing in accordance with Swedish regulations. All participants met these requirements and had between three and more than ten years of experience working in upper secondary school settings. The participating school nurses were employed in both public and independent schools located in areas with varying socioeconomic characteristics and academic orientations.

Participants had between three and more than ten years of experience working in upper secondary school settings and represented both public and independent schools from areas with varying socioeconomic characteristics and academic orientations.

#### Data collection

Data were collected from January to December 2023. Two researchers—one paediatric nurse specialist and one psychiatric nurse specialist—conducted the interviews via Zoom after obtaining written informed consent.

A semi-structured interview guide was developed based on the research team’s clinical and research expertise in paediatric nursing, primary health care nursing, global and public health, psychiatric nursing, and school nursing. Principles for qualitative interviewing described by Kvale and Brinkmann [21] and was deemed satisfactory and therefore included in the analysis informed the development of the interview guide.

Interviews lasted 28–51 min and were transcribed verbatim by the first author.

#### Reflexivity and researcher characteristics

The research team consisted of five authors with complementary professional backgrounds: one with extensive expertise in public health and qualitative methods, two paediatric nurse specialists, one psychiatric nurse specialist with longstanding clinical experience in adolescent mental health, and one nurse specialised in district care. This diversity provided a broad understanding of school health services and adolescent mental health, shaping both data generation and interpretation.

The team acknowledged that their professional assumptions could influence interactions with participants and analytical decisions. Reflexive practices included ongoing discussions about positionality, examination of assumptions during coding, and memo writing throughout analysis. One transcript was jointly coded to calibrate interpretative approaches. Differences in interpretation were discussed openly, allowing professional experiences to serve as an analytical resource while preventing unexamined bias.

#### Data analysis

Reflexive thematic analysis [20], guided the analytic process. One transcript was read and coded by all authors to establish analytic alignment, after which transcripts were divided so that each was read and coded by at least two team members. Codes were developed inductively with the study aim in mind and compared across researchers. Initial codes were iteratively clustered into preliminary themes through team discussions. These were refined through continued analytic engagement until a coherent thematic structure was developed. The final themes are presented in the Findings section.

#### Ethical considerations

Ethical approval was obtained from the Swedish Ethical Review Authority (Etikprövningsmyndigheten), a national government agency under the Swedish Ministry of Education and Research (Ref. No. 2022-06924-01) and the study adhered to the ethical principles outlined in the Declaration of Helsinki [22]. Participants received written and oral information about the study and provided informed consent prior to participation. Participation was voluntary, with the right to withdraw at any time without consequence.

To protect confidentiality, all transcripts were anonymised and identifying details were removed prior to analysis. Audio files and transcripts were stored securely on encrypted, password-protected servers in accordance with institutional data protection procedures. No compensation was provided to the study participants, and the study was assessed as involving minimal risk to participants.

#### Rigor

To enhance trustworthiness, we sought a diverse sample of school nurses representing different school types, program orientations, and socioeconomic contexts, thereby strengthening variation in experiences and information power. In line with reflexive thematic analysis [20], the researchers’ varied clinical and methodological backgrounds were treated as analytical resources rather than sources of bias.

Rigour was supported through an iterative and collaborative analytic process. Codes developed independently were compared and discussed to refine interpretations and develop shared analytic direction. Reflexive memo writing and team discussions throughout the process enhanced transparency and interpretative depth. Rather than striving to “avoid subjectivity,” we engaged reflexively with our disciplinary perspectives, acknowledging how they shaped meaning-making and theme development. This reflexive stance, combined with rich data and transparent analytical decisions, supports the credibility and trustworthiness of the findings.

## Findings

### Theme 1: Fostering mental wellbeing — the school nurse as a trusted ally in a complex world

School nurses play a vital role in identifying and addressing students’ mental health needs. Using multiple approaches, they detect concerns via direct and indirect indicators—ranging from headaches and stomach aches to shifts in body language and affect. As one nurse noted:

“*Many students initially present with physical symptoms such as headaches or stomach aches*,* and it can take time to understand the underlying issues. Some students are extremely stressed and busy*,* and while we can sometimes address the problems early*,* it is not always straightforward*.” — Interview 7.

Collaboration with teachers, students and parents is integral: teachers observe changes in behavioural or performance, parents report signs at home, and students’ self-reports of stress, anxiety, panic, and sleep problems often enable earlier intervention. In addition to referrals and questionnaires, nurses rely on their observations and scheduled health checks, maintaining vigilance for stress and anxiety. Trust, cultivated through ongoing relationships, creates a safe space for disclosure:

“*A large part of my work is about creating a safe environment where students feel comfortable opening. Sometimes*,* it’s as simple as offering them a cup of tea and sitting with them until they feel calmer*.” — Interview 2.

Competing academic demands, sleep disruption, and social pressures were frequently described, alongside broader anxieties related to societal issues. Family dynamics mattered: engaged parents were seen as protective, whereas disengagement could undermine wellbeing. Many nurses kept an open-door policy and created welcoming spaces for rest and conversation; informal encounters were valuable for relationship-building and timely support:

“*It is essential to meet each student based on their unique circumstances*,* particularly in a school with students from diverse cultures and languages*.” — Interview 3.

Nurses also emphasized lifestyle patterns, sleep, activity, and nutrition—as common contributors:

“*It often involves a combination of factors such as lack of sleep*,* insufficient physical activity*,* and irregular eating habits. When students sleep only four hours per night and eat one meal a day*,* it’s no surprise that they struggle to cope with the challenges they face.”* — Interview 6.

Overall, trust-based relationships, collaborative networks, and proactive identification strategies were seen as central to helping students navigate contemporary challenges.

These insights highlight the need for app features that support early recognition of indirect signs of distress, facilitate structured communication, and complement the trust-based relationships central to school nursing practice.

### Theme 2: Nurturing minds — the digital dance of support and solitude

Participants perceived both opportunities and risks in integrating a mobile app into school health support. Potential value included assistance with time management (e.g., reminders for study routines, exercise, or mindfulness), strengthened self-perception through structured habits, and access to a self-care toolkit for stress and anxiety. Apps could facilitate early detection, provide psychoeducational resources and links to professional help, and enable communication via chat, potentially fostering peer support. As one nurse reflected:

*“Students often present in a state of panic*,* overwhelmed by stress or anxiety related to school demands*,* and it becomes evident they need immediate support to manage these pressures.”* — Interview 2.

Digital follow-ups and appointment scheduling were viewed as ways to optimise resources and improve accessibility for students reluctant to seek traditional care. At the same time, nurses flagged concerns about anonymity and secure identification, workload implications, and the risk that digital communication might erode the personal connection of face-to-face care:

*“We face a challenge in addressing the sheer amount of stress students experience*,* and balancing digital tools with in-person care is crucial for not losing the personal connection students often need.”* — Interview 9.

Clear boundaries for availability were deemed essential, with attention to response times and escalation procedures:

*“We can’t be available 24/7 through an app; it’s unrealistic. Students will need to understand that there are limits to our availability.”* — Interview 4.

Some were skeptical about chat-only support, noting that nuanced assessment can be difficult digitally and should be complemented by in-person contacts. Ultimately, nurses viewed mobile apps as promising adjuncts whose success depends on careful implementation—prioritizing usability, ethical safeguards, realistic workload planning, and alignment with relational nursing practice to enhance accessibility and effectiveness of school-based mental health support.

## Discussion

This study positions the proposed mobile app as a relational technology, whose feasibility depends on whether the logic of care—trust, boundaries, and responsiveness—is embedded in governance and everyday workflows. Guided by RTA, we make explicit the theoretical assumptions underpinning our themes to avoid descriptive surface readings and provide a coherent interpretative stance [20]. School nurses reported that, although multiple strategies are already in use, additional accessible tools are needed to facilitate earlier identification and intervention. A mobile app was perceived as promising for lowering barriers to help-seeking, yet concerns about safety, confidentiality, and anonymity must be addressed for responsible implementation in school health services.

### Interpreting needs across contexts: pervasive pressures and cumulative burden

Nurses described adolescents struggling to balance diet, physical activity, schoolwork, leisure, and sleep—patterns seen across diverse socioeconomic settings and aligned with multifactorial determinants of youth mental health [2, 4]. The perceived intensification of these pressures underscores the need for scalable supports in schools. Within this landscape, a digital companion can function as a low-threshold adjunct—not a replacement—to nurse–student relationships, offering structured self-management while maintaining clear pathways to professional care.

### Alignment with emerging evidence on digital youth mental health

Synthesis studies indicate moderate effects for digital interventions in youth, with the strongest signals for CBT-oriented approaches targeting anxiety and depression, alongside consistent cautions regarding engagement, ethics, and long-term outcomes [18, 23]. Evidence specific to adolescents suggests mobile interventions may reduce anxiety symptoms [24], reinforcing the potential of school-embedded tools. Young people with mental illness also express strong interest in supports targeting physical health—especially activity, sleep, and diet—when integrated with mental health tracking and expert-led content [25]. Our feasibility focus complements this literature by specifying workflow and relational conditions under which benefits are most likely to be realised in schools.

### Design features to support everyday regulation and relational care

School nurses prioritised features addressing daily regulation—time management aids, reminder notifications, and toolkits for stress, anxiety, and self-care—aligning with evidence that psychoeducation, coping libraries, goal setting, and prompts support engagement and symptom relief [11]. Interactive monitoring of stress and anxiety was valued to enable proactive follow-up. Framed as relational technology, such features should augment (not bypass) nurse contact, preserve boundaries, and support timely escalation when warning signals appear.

### Ethics, safety, and confidentiality in the Swedish regulatory context

Decisive concerns centered on safety and confidentiality: verifying user identity and protecting sensitive disclosures within stringent legal requirements. As independent healthcare providers, school nurses operate under Swedish regulation governing professional responsibilities, confidentiality, and care quality. Future development should evaluate authentication that balances robust assurance with ease of access, potentially using tiered verification across feature sets (e.g., anonymous self-help vs. identified escalation pathways), in line with prior work on ethical risks in digital youth mental health [16, 17].

### The anonymity paradox: lowering thresholds while enabling safe escalation

Anonymity can reduce stigma and facilitate disclosure—critical given adolescents’ fear of judgement and reluctance to seek help [2, 6, 7, 16]. Yet anonymity becomes problematic when acute risk is disclosed, constraining timely intervention [15, 16]. These findings suggest that a form of graduated anonymity may be useful—for example, allowing anonymous engagement for low-risk self-help and psychoeducation, while requiring identification (or just-in-time identity capture) when high-risk indicators are flagged.

### Timeliness, responsiveness, and the value of reflection

Engagement improves when help feels accessible; however, brief delays can also support reflection and use of personal coping strategies—beneficial for resilience. Delays are risky in crises [15, 16, 26, 27]. Bounded availability and responsive triage is a possibility: [1] clear service hours and escalation protocols; [2] automated just-in-time guidance for non-urgent queries; and [3] immediate crisis pathways for urgent risk. This respects nurses’ workload and relational boundaries while sustaining a sense of access.

### Adoption and implementation: embedding within trusted relationships and workflow

Consistent with technology acceptance models—specifically the Technology Acceptance Model (TAM) and the Unified Theory of Acceptance and Use of Technology (UTAUT)—adoption is strongest when tools are useful, usable, and supported by social influence and facilitating conditions [28, 29]. Embedding digital supports within trusted nurse–student relationships and routine workflows is likely to outperform pure self-help models. To address common barriers (low motivation, time constraints, competing demands), micro-engagement—brief, highly accessible interactions that deliver small wins—may sustain use without overwhelming students or staff [16].

Taken together, our findings point to six feasibility principles: [1] graduated anonymity to reduce stigma while preserving safety; [2] bounded availability and triage to protect workloads and ensure timely support; [3] micro-engagement tools (timers, prompts, check-ins) suited to universal school services; [4] tools that allow students to track aspects of mental and physical health supported by reliable, expert-led content; [5] authentication approaches that could be adjusted to the level of risk and sensitivity of the information; and [6] ways of integrating digital support into existing workflows (e.g., referral pathways, documentation) in ways that are compatible with Swedish data governance and professional standards.

### Strengths and limitations

A key strength is the explicit theoretical framing (relational technology; logic of care) and the RTA approach, which clarifies assumptions and supports coherent interpretation [20]. The qualitative, context-bound design (Swedish school healthcare services) may limit transferability, and the study did not assess clinical efficacy or long-term outcomes. Engagement dynamics and the perceived acceptability of digital support may differ across settings, particularly in jurisdictions with varying data protection requirements, service delivery models, and levels of digital infrastructure, which may influence both implementation and use of digital mental health support.

### Future research

Future work could build on these findings by exploring how elements such as graduated anonymity and authentication can be made usable and acceptable in practice; by examining how digital self-management tools might be combined with nurse-initiated support; and by investigating patterns of engagement and potential equity implications. A natural next step in this project would be to co-develop and refine a prototype together with school nurses to ensure that trust, boundaries, and responsiveness are meaningfully integrated into the design.

## Conclusion

This study highlights school nurses’ central role in identifying and addressing adolescent mental health concerns through trust-based, relational care embedded in students’ everyday school contexts. Mental health needs were often identified indirectly, underscoring the importance of continuity, professional judgement, and low-threshold access within school healthcare services. From the nurses’ perspective, a mobile app may function as a valuable adjunct to existing practice by supporting self-management, improving accessibility, and lowering barriers to help-seeking among adolescents.

However, the findings emphasise that digital tools must complement rather than replace face-to-face care. Ethical considerations—including confidentiality, anonymity, patient safety, and workload—were decisive for acceptability and feasibility. Framing the app as a relational technology, the study identifies key conditions for implementation, such as graduated anonymity, bounded availability, and clear escalation pathways integrated into nurses’ workflows. Overall, the findings suggest that mHealth solutions in school nursing may strengthen early identification and preventive mental health support when grounded in relational care principles and aligned with professional and regulatory frameworks.

## Supplementary Information

Below is the link to the electronic supplementary material.

Supplementary Material 1

Supplementary Material 2

## Data Availability

Due to confidentiality requirements and the sensitive nature of the interview data, transcripts cannot be shared publicly. Anonymised excerpts supporting the findings are included in the article, and additional information can be made available upon reasonable request to the corresponding author.
