# Supplementary material for: School nurses’ perspectives on key features of a mobile app to support early identification of adolescent mental health problems: a qualitative study
Source: BMC Nurs. 2026 Aug 29;25:768. doi: 10.1186/s12912-026-05302-7 (PMC13525641; doi:10.1186/s12912-026-05302-7)
Supplement: Supplementary file 1 [file 12912_2026_5302_MOESM1_ESM.docx]

**Appendix 1**

Interview guide

Describe the school you work at.

How can you as a school nurse discover if a pupil is experiencing stress, worries or depression?

How do pupils express that they are not feeling well?

How do you handle these situations?

What kind of support do you need to help pupils who are experiencing stress, worries or depression?

Do you think you have the aids you need or what else would you need?

Have you ever had a visit from a pupil because they experience stress, worries or depression? How do you handle this conversation?

Do you have experiences of apps that are used to handle stress, worries or depression?

What are your opinions/thoughts of these apps?

If there was an app that you as a school nurse could use to give help and support with mental unhealth to adolescents, what would you think it would contain?

Can you specify what kind of features you would wish for in an app?

What benefits could there be with that sort of app?

What disadvantages could there be with that sort of app?

If this kind of app contained a chat feature where pupils could have contact with you, do you believe it would facilitate to recognize early signs of mental unhealth in pupils? To catch these pupils, what would you write about in the chat?

Do you see any benefits of talking with pupils about mental unhealth in a chat compared to face to face? Could you describe it?

Do you see any disadvantages with talking about mental unhealth with pupils in a chat to face to face? Could you describe it?
