# Supplementary material for: School nurses’ perspectives on key features of a mobile app to support early identification of adolescent mental health problems: a qualitative study
Source: BMC Nurs. 2026 Aug 29;25:768. doi: 10.1186/s12912-026-05302-7 (PMC13525641; doi:10.1186/s12912-026-05302-7)
Supplement: Supplementary file 2 [file 12912_2026_5302_MOESM2_ESM.docx]

**Appendix 2**

**Table 1**

**Overview of the analytic development from initial clusters to final themes**

| **Final Theme** | **Initial analytic clusters (illustrative)** |
| --- | --- |
| **Fostering mental wellbeing: The school nurse as a trusted ally in a complex world** | -Indirect indicators of distress (somatic complaints, behavioural shifts)  -Trust-building and relational continuity  -Collaboration with teachers and parents  - Lifestyle-related contributors (sleep, eating, activity) |
| **Nurturing minds: the digital dance of support and solitude** | - Opportunities for digital self-management  - Communication, accessibility, and early detection  - Concerns about anonymity and confidentiality  -Balancing digital and face-to-face care; maintaining boundaries |
